# Energy and Potassium Ion Homeostasis during Gamma Oscillations

**DOI:** 10.3389/fnmol.2016.00047

**Published:** 2016-06-16

**Authors:** Oliver Kann, Jan-Oliver Hollnagel, Shehabeldin Elzoheiry, Justus Schneider

**Affiliations:** ^1^Institute of Physiology and Pathophysiology, University of HeidelbergHeidelberg, Germany; ^2^Interdisciplinary Center for Neurosciences (IZN), University of HeidelbergHeidelberg, Germany

**Keywords:** cognition, extracellular potassium concentration, GABA-A receptor, membrane ion transport, mitochondria, Na^+^/K^+^-ATPase, neural information processing, tissue oxygen tension

## Abstract

Fast neuronal network oscillations in the gamma frequency band (30–100 Hz) occur in various cortex regions, require timed synaptic excitation and inhibition with glutamate and GABA, respectively, and are associated with higher brain functions such as sensory perception, attentional selection and memory formation. However, little is known about energy and ion homeostasis during the gamma oscillation. Recent studies addressed this topic in slices of the rodent hippocampus using cholinergic and glutamatergic receptor models of gamma oscillations (GAM). Methods with high spatial and temporal resolution were applied *in vitro*, such as electrophysiological recordings of local field potential (LFP) and extracellular potassium concentration ([K^+^]_o_), live-cell fluorescence imaging of nicotinamide adenine dinucleotide (phosphate) and flavin adenine dinucleotide [NAD(P)H and FAD, respectively] (cellular redox state), and monitoring of the interstitial partial oxygen pressure (pO_2_) in depth profiles with microsensor electrodes, including mathematical modeling. The main findings are: (i) GAM are associated with high oxygen consumption rate and significant changes in the cellular redox state, indicating rapid adaptations in glycolysis and oxidative phosphorylation; (ii) GAM are accompanied by fluctuating elevations in [K^+^]_o_ of less than 0.5 mmol/L from baseline, likely reflecting effective K^+^-uptake mechanisms of neuron and astrocyte compartments; and (iii) GAM are exquisitely sensitive to metabolic stress induced by lowering oxygen availability or by pharmacological inhibition of the mitochondrial respiratory chain. These findings reflect precise cellular adaptations to maintain adenosine-5′-triphosphate (ATP), ion and neurotransmitter homeostasis and thus neural excitability and synaptic signaling during GAM. Conversely, the exquisite sensitivity of GAM to metabolic stress might significantly contribute the exceptional vulnerability of higher brain functions in brain disease.

## Gamma Oscillations and Higher Brain Functions

Neuronal information processing is primarily executed by principal cells, such as granule and pyramidal neurons that release excitatory neurotransmitter, glutamate (Bliss and Lømo, 1973; Miles and Wong, 1987; LoTurco et al., 1988; Malenka et al., 1989). It is generally thought that these projection neurons process, transfer, store and retrieve information and therefore, underlie the emergence of higher brain functions such as sensory perception, attentional selection, motor behavior, and memory formation (Buzsáki, 2006; Kullmann and Lamsa, 2007; Hájos and Paulsen, 2009; Ho et al., 2011).

Neuronal information processing, however, depends on the coordination of principal cell activity in cortical networks (Buzsáki, 2006; Traub and Whittington, 2010). Such coordination can be provided by neuronal network oscillations that show a wide spectrum of frequencies, ranging from about 0.05 Hz to 600 Hz (Buzsáki and Draguhn, 2004). Prominent examples are network oscillations in the theta (4–12 Hz), beta (13–30 Hz) and gamma (30–100 Hz) bands, which are associated with different cognitive and behavioral states (Buzsáki, 2006; Hájos and Paulsen, 2009; Uhlhaas and Singer, 2010; Watrous et al., 2015). Of course, this does not preclude the importance of slower oscillations for higher brain functions (Buzsáki, 2006; Schroeder and Lakatos, 2009).

Gamma oscillations (GAM) (30–100 Hz) have been found in many mammalian brain regions, such as visual, auditory, somatosensory and motor systems, and in the hippocampus (Kreiter and Singer, 1992; Murthy and Fetz, 1992; Franowicz and Barth, 1995; Lebedev and Nelson, 1995; Whittington et al., 1995; Gray and Viana Di Prisco, 1997). GAM are associated with rhythmic fluctuations of the membrane potential of 5–10 mV in excitatory pyramidal cells and fast-spiking inhibitory interneurons, reflecting precisely timed incidence of excitatory postsynaptic currents (EPSCs) and inhibitory postsynaptic currents (IPSCs) (Whittington et al., 1995; Penttonen et al., 1998; Fischer et al., 2002; Salkoff et al., 2015). These rhythmic fluctuations support the synchronized generation of action potentials (neuronal “spiking”) in principal cells with great precision (Buzsáki, 2006; Hájos and Paulsen, 2009; Watrous et al., 2015) and thus, permit the coordinated activation of defined sets of neurons, i.e., functional ensembles that are thought to represent the information-carrying multicellular subsets of neuronal networks (Buzsáki and Chrobak, 1995; Whittington et al., 1997; Fries et al., 2007; Traub and Whittington, 2010). GAM have a role in higher brain functions, such as voluntary movement, visual and auditory perception, attentional selection as well as memory formation (Gray et al., 1989; Pantev et al., 1991; Paulsen and Moser, 1998; Haenschel et al., 2000; Melloni et al., 2007; Montgomery and Buzsáki, 2007; Cheyne et al., 2008; Lisman and Buzsáki, 2008; van Vugt et al., 2010; Zhang et al., 2012; Popa et al., 2013). GAM *in vivo* occur transiently on the 100 ms time scale upon sensory input (Pantev et al., 1991; Bragin et al., 1995; Franowicz and Barth, 1995). In the human brain and dependent on the task, however, they can last for prolonged times in the range of minutes (Lehmann et al., 2001; Lutz et al., 2004). A summary of some key features of cortical GAM is given in Table 1.

**Table 1:** **Features of cortical gamma oscillations (GAM)**.

Features of gamma oscillations	Reference
Presence in most cortical areas	Murthy and Fetz (1992); Haenschel et al. (2000); van Vugt et al. (2010); and Popa et al. (2013)
Strong relationship to higher brain functions	Gray et al. (1989); Pantev et al. (1991); Lutz et al. (2004); and Zhang et al. (2012)
Fast rhythmic inhibition by interneurons	Traub et al. (2001); Hájos et al. (2004); Cardin et al. (2009); and Gulyás et al. (2010)
High oxygen consumption rate	Niessing et al. (2005); Kann et al. (2011); and Huchzermeyer et al. (2013)
Increase in [K^+^]_o_ of <0.5 mmol/L	Huchzermeyer et al. (2008); and Kann et al. (2011)
Exquisite sensitivity to metabolic stress	Huchzermeyer et al. (2008); Hájos et al. (2009); Barth and Mody (2011); and Whittaker et al. (2011)

*GAM (30–100 Hz) have been related to sensory perception, attentional selection, motor behavior, and memory formation. These oscillations strongly depend on fast-spiking GABAergic interneurons, such as parvalbumin-positive basket cells, that exert fast rhythmic perisomatic inhibition on principal cells in local networks of the hippocampus and the neocortex. Only some key references are given. See details in the text*.

## Investigating Gamma Oscillations *In Vitro*: Experimental Models and Methods

### Experimental Models in Hippocampal Slice Preparations

GAM in cortical tissue *in vitro* can be reliably induced by various methods, such as electrical stimulation or bath application of cholinergic or glutamatergic receptor agonists. In many studies, acute slices or organotypic slice cultures of the hippocampus have been used (Whittington et al., 1995; Fisahn et al., 1998; Hájos et al., 2009; Kann et al., 2011). An overview about the induction and features of GAM under various recording conditions, i.e., in different models, is given in Table 2. Pharmacologically induced hippocampal GAM *in vitro* share many features with GAM *in vivo*, such as intrinsic generation of GAM in the CA3 region, reversal of the phase of the local field potential (LFP) between stratum pyramidale (cell body layer of pyramidal cells) and stratum radiatum (apical dendritic compartment) of CA3, similar current source density profiles, and highest spiking probability of pyramidal cells at the negative peak of oscillation cycles (CA3, stratum pyramidale) that is followed by spiking of perisomatic inhibitory interneurons within 2 ms, consistent with monosynaptic excitation (Bragin et al., 1995; Penttonen et al., 1998; Csicsvari et al., 2003; Hájos et al., 2004; Hájos and Paulsen, 2009). However, in the majority of *in vitro* studies GAM are persistent for tens of minutes, show a frequency around 40 Hz, and are rarely associated with an additional, slower network rhythm (Table 2).

**Table 2:** **Features of hippocampal gamma oscillations (GAM) *in vitro***.

Slice type	Animal	Age	Recording condition	Energy substrate	Induction	Oxygen fraction	Frequency	Duration	Temperature	Reference
Culture	Rat	p6, div21–42	Submerged	Glucose 5.6 mM	mAChR agonist	95%	40 Hz	Persistent with theta	32°C	Fischer et al. (2002)
Culture	Rat	p7–9, div5–9	Interface	Glucose 10 mM	mAChR agonist	20%	49 Hz	Persistent	34 ± 1°C	Huchzermeyer et al. (2013)
Culture	Rat	p7–9, div7–21	Interface	^†^	mAChR agonist	95%	40 Hz	Persistent	34 ± 1°C	Galow et al. (2014)
Culture	Rat	p7–9, div7–28	Interface	Glucose 10 mM	mAChR agonist/GluR agonist	95%	*24–52 Hz	Persistent	28–36°C	Schneider et al. (2015)
Acute	Rat	adult	Interface	Glucose 10 mM	Electrical stimulation/PE of GluR agonists	95%	40 Hz	Transient	36°C	Whittington et al. (1995)
Acute	Rat	p15–25/adult	Interface	Glucose 10 mM	mAChR agonist	95%	39 Hz	Persistent with theta	34°C	Fisahn et al. (1998)
Acute	Rat	p20–30	Submerged	Glucose 10 mM	^†^mAChR agonist	95%	38–61 Hz	Transient with theta, delta or none	31–32°C	Fellous and Sejnowski (2000)
Acute	Rat	adult	Interface	Glucose 10 mM	PE of GluR agonist	95%	^†^	Transient	35.8 ± 0.5°C	Pöschel et al. (2002)
Acute	Mouse	p18–25	Submerged	Glucose 10 mM	PE of GluR agonist	95%	33 Hz	Transient	29°C	Gloveli et al. (2005)
Acute	Rat	p13–20	Submerged	Glucose 10 mM	mAChR agonist	95%	28 Hz	Persistent with beta	29–33°C	Oren et al. (2006)
Culture/acute	Rat/mouse	p7–9, div7–12/p20–30/adult	Interface/submerged	Glucose 10 mM	mAChR agonist	95%	29–42 Hz	Persistent	34 ± 1°C/25 ± 1°C	Kann et al. (2011)
Acute	Rat	p42–56	Interface	Glucose 10 mM	^†^mAChR agonist/ACh esterase inhibitor	95%	30–47 Hz	Persistent	36 ± 0.5°C	Hollnagel et al. (2015)
Acute	Rat	p2–5/p6–21	Modified interface	Glucose 11 mM	GluR agonist	95%	−/24–35 Hz	Persistent/transient with slow waves	33.5 ± 34°C	Tsintsadze et al. (2015)

Acute slices have a thickness of around 400 μm; the residual thickness of organotypic hippocampal slice cultures is around 200 μm. In most studies, muscarinic acetylcholine receptors (mAChR) were activated with acetylcholine or carbachol, glutamate receptors (GluR) with glutamate or kainic acid. The frequency of GAM also varies with temperature, i.e., ~3.5 Hz increase per degree Celsius for the range of 28–36°C (Schneider et al., 2015). *4 Hz increase when 2 mmol/L lactate was added to 5 mmol/L glucose at 34 ± 1°C. ^†^Various substrates, concentrations or frequencies. div, days in vitro; PE, pressure ejection; p, postnatal day.

The synaptic mechanisms that underlie the generation of GAM have been reviewed in detail, and they largely depend on the ratio of neuronal excitation and inhibition (Bartos et al., 2007; Hájos and Paulsen, 2009). In the cortex, synaptic inhibition is mainly mediated by neurotransmitter, gamma-aminobutyric acid (GABA) that is released from the heterogeneous group of GABAergic interneurons (Mann and Paulsen, 2007; Klausberger and Somogyi, 2008; Fritschy and Panzanelli, 2014; Kaila et al., 2014). Notably, the transient activation of GABA-A receptors has a key role for the generation of GAM. This is because GAM are completely blocked by GABA-A receptor antagonist, bicuculline in various *in vitro* models, and studies also using transgenic mice show that synaptic excitation of fast-spiking, parvalbumin-positive interneurons is required for the generation of normal GAM *in vitro* and *in vivo* (Whittington et al., 1995; Fuchs et al., 2007; Cardin et al., 2009; Sohal et al., 2009; Gulyás et al., 2010; Korotkova et al., 2010; Oren et al., 2010). During hippocampal GAM, individual pyramidal cells generate action potentials at 1–3 Hz *in vitro* and *in vivo*, whereas fast-spiking perisomatic interneurons generate action potentials phase-coupled at almost every gamma cycle (Csicsvari et al., 2003; Hájos et al., 2004; Kann et al., 2014).

### Recordings of LFP, [K^+^]_o_, pO_2_ and Redox State

The LFP, which is also known as micro-, depth or intracranial electroencephalogram (EEG), has been frequently used to monitor neuronal network oscillations *in vitro* and *in vivo*. LFP electrodes are small-sized, have usually a resistance of about 1–2 MOhm and are positioned in the extracellular space. The recorded extracellular potentials arise from all transmembrane ionic fluxes that underlie cellular electrical events, ranging from fast action potentials and postsynaptic potentials in neurons to slow membrane potential fluctuations in glial cells (Buzsáki et al., 2012; Einevoll et al., 2013; Hales and Pockett, 2014). Recent estimates suggest that >95% of the LFP originates in the vicinity of about 250 μm of the electrode tip (Katzner et al., 2009). Thus, the LFP recording represents a spatial average of all electrical events in a confined volume of neuronal tissue at a given point in time. Although still under debate, the prominent influence of tip geometry and impedance has not been proven yet (Nelson and Pouget, 2010).

The [K^+^]_o_ can be determined with double-barreled microelectrodes in neuronal tissue. The reference barrel (LFP) is filled with 154 mM NaCl solution, and the ion-sensitive barrel with an ion-exchanger (K^+^ ionophore cocktail) and 100 mM KCl ([K^+^]_o_; Heinemann and Lux, 1975; Gorji and Speckmann, 2009; Papageorgiou et al., 2016). Recordings of [K^+^]_o_ have been used to determine the level of neuronal activation and K^+^-homeostasis, including the functions of glial cells (see below). K^+^-sensitive microelectrodes measure the accumulation of K^+^ in a restricted extracellular space, irrespective of whether K^+^ is released from dendrites, somata or axons. The microelectrodes detect changes in [K^+^]_o_ from the surrounding tissue microenvironment of less than 100 μm for most conditions of experimental K^+^-electrophoresis or electrical stimulation (Lux, 1974; Heinemann et al., 1986; Lux et al., 1986; Kann et al., 2003a,b).

The interstitial partial oxygen pressure (pO_2_) or the oxygen concentration in neuronal tissue can be determined with Clark-type oxygen microsensors, which are polarographic electrodes. Because oxygen is the final electron acceptor at the mitochondrial respiratory chain, oxygen consumption provides a valuable indirect measure of the metabolic rate in tissues (Rolfe and Brown, 1997). During the recording, oxygen diffuses from the adjacent tissue through a silicone membrane at the sensor tip and is reduced at a gold cathode within the microsensor. The resulting current is measured with a picoammeter and converted into mmHg (or mmol/L) according to calibration curves (Revsbech, 1989; Lecoq et al., 2009; Thomsen et al., 2009). Oxygen microsensors measure quite locally, i.e., with a spatial resolution of 1–2 times the outside tip diameter (8–12 μm). Oxygen consumption rates (mmol/L per min) can be calculated by recording pO_2_ depth profiles in slice preparations and by applying mathematical models that consider convective transport, diffusion, and activity-dependent oxygen consumption (Hall and Attwell, 2008; Huchzermeyer et al., 2013).

The cellular redox state is a useful and non-invasive tool to get insight into neuronal energy metabolism. For this purpose, live-cell fluorescence imaging of nicotinamide adenine dinucleotide (phosphate) and flavin adenine dinucleotide [NAD(P)H and FAD, respectively] have been applied. These dinucleotides serve in cellular energy transfer and have been used to get insight into activity-dependent changes in cytosolic and mitochondrial redox state, and thus adaptations in energy metabolism (Kann et al., 2003a,b; Shuttleworth et al., 2003; Kasischke et al., 2004; Brennan et al., 2006; Ivanov et al., 2011). When excited with ultraviolet light the reduced forms (NADH and NADPH) are fluorescent, while the oxidized forms are non-fluorescent. Investigators often refer to changes in NAD(P)H fluorescence because the emission spectra of NADPH and NADH overlap, and their redox states are coupled via nicotinamide nucleotide transhydrogenase (Schuchmann et al., 2001; Kann and Kovács, 2007). Cellular NAD(P)H fluorescence is primarily governed by activities of the respiratory chain (electron transport chain) and the tricarboxylic acid (TCA) cycle in mitochondria. However, relative changes in NAD(P)H fluorescence in brain slices are influenced by a variety of additional factors and need careful interpretation (Kann and Kovács, 2007; Berndt et al., 2015). Electron transport flavoproteins and α-lipoamide dehydrogenase contribute to about 75% of the flavin fluorescence in neurons (Kann and Kovács, 2007). Because both of them are also closely linked to the mitochondrial NADH pool FAD fluorescence provides insight into the mitochondrial redox state (Shuttleworth et al., 2003; Kann and Kovács, 2007). Because here the oxidized form is fluorescent, changes in FAD fluorescence are opposite to NAD(P)H fluorescence. In most studies using epifluorescence recordings (epi-illuminating light source and CCD camera), the changes in NAD(P)H fluorescence originate from neuronal and glial compartments of virtually all layers (*z*-axis) of the slice (Shuttleworth et al., 2003; Foster et al., 2005; Kann and Kovács, 2007; Huchzermeyer et al., 2008).

## Energy Metabolism During Gamma Oscillations

Recent experimental studies have started to define the bioenergetics of cortical GAM. The available experimental evidence from many *in vitro* and some *in vivo* studies in animals and humans indicates that GAM in the hippocampus and the neocortex are associated with significant cellular adaptations to maintain energy, ion and neurotransmitter homeostasis and thus neuronal excitability and synaptic signaling. Below, we discuss the homeostatic adaptations underlying GAM, with emphasis on activity-dependent changes in pO_2_, cellular redox state [NAD(P)H and FAD] and [K^+^]_o_.

Recordings of LFP and pO_2_ with high temporal and spatial resolution revealed a positive correlation between the power of GAM and the local decrease in pO_2_ in acute slices and slice cultures of the hippocampus, reflecting the increase in activity-dependent oxygen consumption (Kann et al., 2011). Intriguingly, the local decrease in pO_2_ during GAM was significantly larger compared to repetitive electrical stimulation (10 s, 20 Hz) and close to the decrease in pO_2_ associated with pathological activity, i.e., seizure-like events. These findings were supported by a second study in slice cultures of the rat hippocampus demonstrating the about twofold increase in local oxygen consumption rate during GAM (about 11 mmol/L per min) compared with spontaneous asynchronous neuronal network activity (about 5 mmol/L per min). This follow-up study was based on depth profiles of local pO_2_ with high spatial resolution and mathematical modeling of convective transport, diffusion and activity-dependent consumption of oxygen (Huchzermeyer et al., 2013). Notably, the oxygen microsensor measured locally in stratum pyramidale, i.e., the layer where the somata of pyramidal cells are densely packed and mainly receive strong perisomatic inhibition from the complex axon arbors of GABAergic parvalbumin-positive basket cells (Sik et al., 1993; Hu et al., 2014; Kann et al., 2014). Thus, the data reflect oxygen consumption related to postsynaptic inhibition and action potential generation in pyramidal cells as well as axonal action potentials and fast rhythmic release of GABA from basket cells (Kann et al., 2014). Further experimental studies are required to determine the fractions of energy utilization in pyramidal cells (perisomatic postsynaptic potentials and action potentials at the axon initial segment) vs. GABAergic basket cells (action potentials and GABA release in the complex axon arbor).

The oxygen consumption rates during persistent GAM are higher than those obtained during spontaneous asynchronous activity or repetitive electrical stimulation in acute hippocampal and cerebellar slices reported to range from 0.7 to 1.3 mmol/L per min (Hall and Attwell, 2008; Ivanov and Zilberter, 2011; Hall et al., 2012). Several aspects need to be considered when comparing oxygen consumption rates from different experimental studies *in vitro* and *in vivo*. The high oxygen consumption rate (Huchzermeyer et al., 2013) was determined during agonist-induced persistent GAM in the pyramidal cell layer (perisomatic region) of the CA3 network in slice cultures that feature high tissue preservation and connectivity (Zhao et al., 2012; Studer et al., 2014; Schneider et al., 2015), thus reflecting a high activity state. In addition, the local CA3 network is a generator of GAM of high power within the hippocampus, and the electrophysiological and bioenergetical features of this network may not entirely apply to other neuronal networks or cortical regions (Buzsáki, 2006; Traub and Whittington, 2010; Kann et al., 2014). Another aspect is the spatial resolution. “Local pO_2_” in the depth profiles as determined with oxygen microsensors in slice preparations refers to a distance of about 0.2 mm (Hall and Attwell, 2008; Huchzermeyer et al., 2013). For comparison, the resolution of functional magnetic resonance imaging (fMRI) technologies *in vivo* is in the order of 1 mm^3^ and more. However, the *in vitro* studies suggest that GAM are associated with high energy expenditure.

These findings are in line with studies in humans using positron emission tomography with 2-deoxy-2-[18F] fluoro-D-glucose. The studies demonstrated stimulus-dependent increases in glucose metabolic rate in primary and associative visual cortices of about 40% and 60%, respectively (Phelps et al., 1981) as well as a positive correlation between spectral amplitudes of GAM and the regional glucose uptake, which was determined during seizure-free intervals in patients with non-lesional focal epilepsy (Nishida et al., 2008). Remarkably, the correlations between glucose uptake and oscillations in other human frequency bands, such as theta (4–7 Hz), alpha (8–12 Hz) and beta (16–32 Hz) were found to be either minor or absent. The tight correlation between GAM and energy expenditure is supported by studies using fMRI as a measure of neurovascular coupling. The power of GAM positively correlated with the hemodynamic fMRI response in the cat visual cortex (Niessing et al., 2005). Positive correlations between GAM and fMRI signals were also described for the human cortex during specific tasks and for nearly the entire cerebral cortex of the monkey (Lachaux et al., 2007; Schölvinck et al., 2010; Scheeringa et al., 2011). In general, there appears to be a linear relationship between neuronal activity, energy metabolism and hemodynamic responses (Sheth et al., 2004; Martin et al., 2006; Viswanathan and Freeman, 2007; Hyder et al., 2013). However, details about the relationships between GAM in local networks, energy expenditure and adaptations in blood flow need to be defined in future studies (Sumiyoshi et al., 2012).

Recently, the utilization of various energy substrates during persistent GAM was explored in rat slice cultures. It was demonstrated that GAM can be powered by various energy-rich substrates, with glucose being most effective (Galow et al., 2014). Notably, the high concentration (20 mmol/L) of either lactate or pyruvate was necessary to maintain GAM. The amplitude of the GAM, however, was significantly reduced. In another study, the addition of lactate (2 mmol/L) to lowered glucose concentration (5 mmol/L) exclusively increased the frequency by about 4 Hz, whereas the power of GAM was unchanged (Schneider et al., 2015). Therefore, lactate appears to be much less beneficial to fuel fast neuronal network oscillations compared with neuronal activity that was evoked by electrical stimulation in slice preparations (Schurr et al., 1988; Izumi et al., 1997; Schurr and Payne, 2007; Ivanov et al., 2011; Barros, 2013).

**Figure 1:**
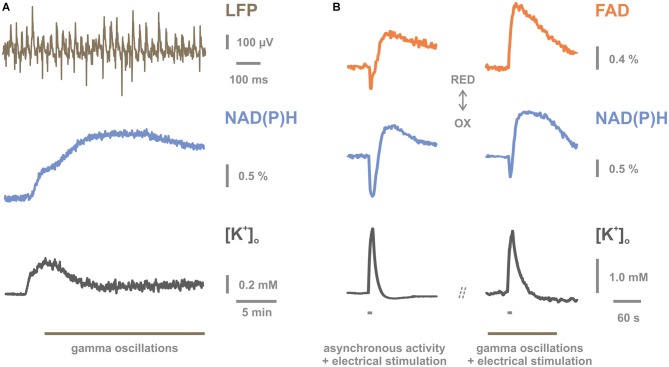
**Mitochondrial redox state during gamma oscillations (GAM) *in vitro*. (A)** Persistent GAM at around 40 Hz are generated in the presence of acetylcholine and physostigmine in the CA3 region of rat hippocampal slice cultures as revealed by local field potential (LFP) recordings (upper trace). Persistent GAM are associated with a long-lasting elevation in nicotinamide adenine dinucleotide (phosphate) (NAD(P)H) fluorescence (middle trace). This significant shift in the redox state towards reduction likely reflects the activation of reducing processes (tricarboxylic acid cycle and glycolysis) and/or the limitation of oxidizing processes (mitochondrial respiratory chain). Persistent GAM (symbolized by gray line) are associated with a fluctuating elevation in [K^+^]_o_ of less than 0.2 mmol/L (lower trace). Note the higher temporal resolution of the upper trace illustrating GAM. **(B)** Simultaneous recordings of flavin adenine dinucleotide (FAD) (upper traces) and NAD(P)H (middle traces) fluorescence and [K^+^]_o_ (lower traces) during spontaneous asynchronous activity (left) and persistent GAM (right). Recordings were made at identical locations during the two network activity states in a given slice culture. Superimposed repetitive electrical stimulation (10 s, 20 Hz, gray bars) resulted in FAD and NAD(P)H fluorescence transients with biphasic shapes relative to pre-stimulation baseline as well as transient changes in [K^+^]_o_. Upward deflections in fluorescence traces indicate shifts in the redox state towards reduction (gray arrow, RED), and downward deflections towards oxidation (gray arrow, OX; “REDOX” state). Biphasic transients are illustrated such that downward deflections in FAD and NAD(P)H indicate increase and decrease in emission fluorescence intensity, respectively. Note that amplitudes and kinetics of downward deflections in FAD and NAD(P)H fluorescence are clearly altered during GAM (right), likely indicating near-limit utilization of mitochondrial oxidative capacity. The K^+^-sensitive microelectrode was placed in stratum pyramidale, i.e., the layer where the somata of pyramidal cells are densely packed and receive strong perisomatic inhibition from the complex axon arbors of basket cells. Note that the decay time of [K^+^]_o_ transients is prolonged during GAM (right). See details in the text. This figure is reproduced and modified from Kann et al. (2011) (Figures 5C, 7A,C) by permission of Oxford University Press on behalf of The Guarantors of Brain. This material is published under a Standard License and is not covered by any Creative Commons License. For commercial and non-commercial reuse, please, seek the permission of journals.permissions@oup.com.

Using FAD and NAD(P)H fluorescence imaging during GAM, the concomitant changes in the mitochondrial redox state were investigated in the CA3 network of hippocampal slice cultures. GAM were associated with a shift towards reduction of the dinucleotides although the interstitial pO_2_ was hyperoxic (Huchzermeyer et al., 2008; Kann et al., 2011; Figure 1A). This finding might reflect an increase in the availability of energy-rich carbon molecules as a result of enhanced glycolysis in neuronal and astrocytic compartments (Kasischke et al., 2004; Brennan et al., 2006; Hertz et al., 2007; Galow et al., 2014) or an imbalance in the activities of neuronal TCA cycle and mitochondrial respiratory chain. Moreover, repetitive electrical stimulation (20 Hz, 10 s) superimposed on GAM resulted in significantly smaller shifts towards oxidation of the dinucleotides compared to controls (Kann et al., 2011; Figure 1B). This suggests that GAM are associated with near-limit utilization of mitochondrial oxidative capacity and provides further evidence for the high energy expenditure during GAM. The data might also imply that rapid and sufficient supply of oxygen and nutrients through changes in blood flow is a fundamental prerequisite for the maintenance of ion and energy homeostasis and therefore, the capability of local neuronal networks to express fast oscillations (Scheeringa et al., 2011; Sumiyoshi et al., 2012; Huchzermeyer et al., 2013; Kann et al., 2014). However, prolonged performance of neuronal mitochondria carries an inherent risk of increased generation of superoxide anion at complexes I and III of the mitochondrial respiratory chain (Morán et al., 2012; Kann, 2016). Increased superoxide anion levels can favor the accumulation of hydrogen peroxide and the generation of other reactive oxygen and nitrogen species (ROS and RNS, respectively). Some of them are reactive molecules with high biological toxicity owing to the capability to oxidize macromolecules such as lipids, DNA, and proteins (Kann and Kovács, 2007; Morán et al., 2012). In particular, fast-spiking, parvalbumin-positive basket cells might transiently generate high ROS levels because the unique electrophysiological and bioenergetical characteristics may frequently result in mismatching changes of metabolic state, Ca^2+^-load and pO_2_ in mitochondria (Kann, 2016). Such mismatches have been discussed to promote the generation of superoxide anion (Kann and Kovács, 2007; Nicholls, 2008; Adam-Vizi and Starkov, 2010). Detailed experimental studies are required to determine free radical generation and oxidative stress in excitatory and inhibitory neurons during GAM.

Intriguingly, the electrophysiological and bioenergetic features of the hippocampal CA3 network, i.e., highest levels in gamma oscillation power, oxygen consumption, and mitochondrial performance, also correlated with the highest expression of complex I subunits (Kann et al., 2011; Wirtz and Schuelke, 2011). Complex I (NADH:ubiquinone oxidoreductase) is a member of the respiratory chain in mitochondria. It is composed of up to 46 subunits that are encoded by nuclear and mitochondrial DNA (Distelmaier et al., 2009). Complex I has been discussed to strongly control oxidative phosphorylation in mitochondria, and to be involved in the pathogenesis of various neurodegenerative diseases (Pathak and Davey, 2008; Koopman et al., 2013). The pattern of complex I gene expression in the hippocampus might reflect unique enzymatic properties of neuronal mitochondria in the CA3 network to meet the homeostatic challenges that are associated with the generation of GAM (Kann, 2012).

Conversely, a variety of studies demonstrated the fast decline of GAM in hippocampal slice preparations during metabolic stress, i.e., (i) by lowering the oxygen fraction in the ambient atmosphere to the normoxic range in the semi-interface recording condition (Huchzermeyer et al., 2008); (ii) by lowering the application rate of oxygenated recording solution in the submerged recording condition (Hájos et al., 2009); and (iii) by induction of hypoxic events (Fano et al., 2007; Pietersen et al., 2009). The fast decline of GAM was also found during pharmacological inhibition of the respiratory chain by rotenone (acting on complex I) or potassium cyanide (acting on cytochrome *c* oxidase in complex IV), and in the presence of protonophores that exert mitochondrial membrane uncoupling (Kann et al., 2011; Whittaker et al., 2011). Moreover, the exquisite sensitivity of GAM to mitochondrial dysfunction has been identified because other types of neuronal activity, such as electrical stimulus-evoked neuronal activation and pathological seizure-like events were more resistant to both, respiratory chain inhibition and low interstitial pO_2_ (Huchzermeyer et al., 2008; Kann et al., 2011). Similar observations were reported for unilateral hippocampal ischemia *in vivo* (Barth and Mody, 2011). These studies consistently show that hippocampal GAM are rapidly impaired during metabolic stress and are in line with data on high energy expenditure during GAM. Mechanistically, dysfunction of fast-spiking GABAergic interneurons, such as parvalbumin-positive basket cells, might be mainly responsible for this rapid impairment. This is likely because these inhibitory interneurons: (i) are crucial for the generation of GAM; and (ii) show unique bioenergetic, biophysical and electrophysiological properties (Kageyama and Wong-Riley, 1982; Gulyás et al., 2006; Hu and Jonas, 2014; Takács et al., 2015). Further details were recently summarized and discussed in reviews about the “interneuron energy hypothesis” (Kann et al., 2014; Kann, 2016).

## Potassium Ion Homeostasis During Gamma Oscillations

The high energy expenditure of neurons during GAM is most likely caused by increased rates of action potentials and postsynaptic potentials. In particular, the significant and widespread increase in rates of EPSPs and IPSPs in the local network elicits strong ion fluxes across the neuronal membrane of both excitatory principal cells and inhibitory interneurons (Wong-Riley, 1989; Hájos and Paulsen, 2009; Kann et al., 2014). These ion fluxes tend to dissipate the gradients of sodium, calcium, potassium and chloride ions, ultimately utilizing potential energy. In order to keep homeostasis and neuronal excitability and thus, ensure maintenance of neuronal information processing, these ionic gradients have to be continuously restored by ion pumps, such as Na^+^/K^+^-ATPase and Ca^2+^-ATPase, and transporters, such as Na^+^/Ca^2+^-exchanger, Na^+^/H^+^-exchanger, and K^+^/Cl^−^-cotransporter (Somjen, 2002; Buzsáki et al., 2007; Kann et al., 2014). In addition, synthesis, release and uptake of neurotransmitters and precursors require various transport processes across neuronal and glial membranes (Bak et al., 2006; Roth and Draguhn, 2012). These transport processes are ultimately powered by cellular energy carrier, adenosine-5′-triphosphate (ATP) that is generated to a large extent by oxidative phosphorylation in neuronal mitochondria (Attwell and Laughlin, 2001; Erecińska and Silver, 2001).

**Figure 2:**
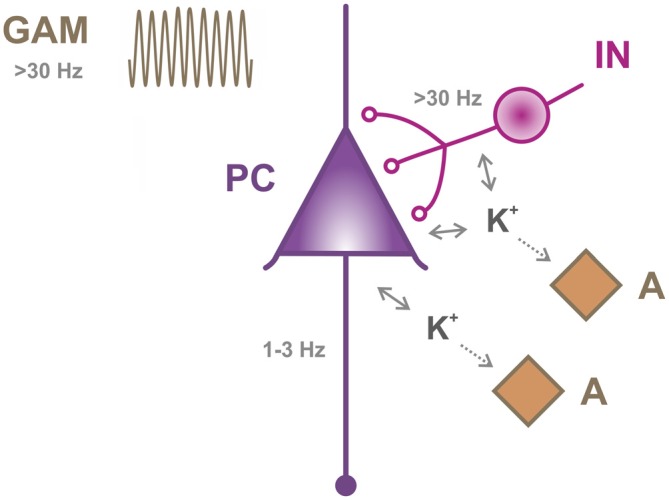
**K^+^-homeostasis during gamma oscillations (GAM) *in vitro*.** The schematic representation illustrates key features of GAM (30–100 Hz) in stratum pyramidale of hippocampal slice preparations. GAM require precise synaptic interactions of excitatory principal cells, such as pyramidal cells (PC), and fast-spiking, inhibitory interneurons (IN), such as parvalbumin-positive basket cells, which feature complex axon arbors. In the hippocampus, fast-spiking interneurons exert fast rhythmic inhibition on the perisomatic region of pyramidal cells by phasic release of GABA from presynaptic terminals (open circles). Interactions with other subtypes of interneurons and excitation of interneurons by pyramidal cells are not illustrated. During GAM, enhanced K^+^-release in stratum pyramidale occurs at several neuronal sites: (i) axons of interneurons and pyramidal cells that fire action potentials at different frequencies; (ii) presynaptic endings of interneurons; and (iii) perisomatic postsynaptic membranes of pyramidal cells. The K^+^-uptake occurs mainly in neurons and might be supported by adjacent astrocytes (A). Note that [K^+^]_o_ does not exceed 3.5 mmol/L during GAM. Note that GAM are associated with a high oxygen consumption rate, indicating adaptations of adenosine-5′-triphosphate (ATP) generation in mitochondria to power ion pumps and secondary ion transport. See details in the text.

However, experimental studies that explored the ion homeostasis during naturally occurring, fast neuronal network oscillations are rare. The few available studies focused on changes in [K^+^]_o_ in hippocampal slice preparations (see below). [K^+^]_o_ was monitored by double-barreled microelectrodes (see above). [K^+^]_o_ is normally between 2.7 to 3.8 mmol/L in neuronal tissue *in vivo*, which is lower than in the other extracellular fluids of the body (Prince et al., 1973; Lux, 1974; Heinemann and Lux, 1977; Somjen, 2002; Gorji and Speckmann, 2009). Keeping [K^+^]_o_ in this narrow range protects central neurons from undue influences on excitability and synaptic transmission because the elevation in [K^+^]_o_ generally depolarizes neuronal membranes, and substantial [K^+^]_o_ elevation has drastic consequences, such as the pathological occurrence of epileptic activity (Zuckermann and Glaser, 1968; Heinemann et al., 1986; Janigro et al., 1997; Somjen, 2002; Jandová et al., 2006; Steinhäuser et al., 2012). Normally, the generation of action potentials and postsynaptic potentials results in K^+^-release from neurons, e.g., through voltage-gated K^+^-channels and non-selective cation channels (Somjen, 2002; Buzsáki et al., 2007; Figure 2). At inhibitory synapses, K^+^-release can be evoked by pre- and postsynaptic GABA-B receptors that open K^+^-channels. In addition, at the postsynaptic membrane K^+^-release drives the extrusion of chloride through K^+^/Cl^−^-cotransporters, such as KCC2, to reverse the Cl^−^-influx that underlies the hyperpolarizing action of GABA-A receptors (Mann and Paulsen, 2007; Blaesse et al., 2009; Kaila et al., 2014). The activation of GABA-A receptors, however, might also cause shunting inhibition in both principal cells and interneurons (Alger and Nicoll, 1979; Andersen et al., 1980; Bartos et al., 2007; Mann and Paulsen, 2007). K^+^-uptake occurs mainly through Na^+^/K^+^-ATPase of neurons and astrocytes. Under certain conditions, it might be supported by astrocytic K^+^-transporters, K^+^-channels and gap-junctions that permit K^+^-buffering and spatial redistribution within the astrocyte syncytium (Somjen, 2002; Steinhäuser et al., 2012). There is first evidence for the role of astrocytes in GAM because functional manipulation of astrocytes markedly decreased the EEG power in the gamma frequency band in awake-behaving mice, whereas neuronal synaptic activity remained intact. The reduction in cortical GAM was accompanied by altered behavioral performance in the novel object recognition test (Lee et al., 2014). However, the details about the nature of the role of astrocytes, for example, in ion and/or neurotransmitter homeostasis, are less clear and require further experimental studies.

Changes in [K^+^]_o_ associated with GAM were explored in the CA3 network of hippocampal slice cultures (Figure 2). The ion-sensitive microelectrodes measured [K^+^]_o_ locally in stratum pyramidale, i.e., the layer where the somata of pyramidal cells are densely packed and receive strong perisomatic inhibition from the complex axon arbors of basket cells (Kann et al., 2014). It was shown that the pharmacological induction of GAM by bath application of acetylcholine at low micromolar concentrations evoked an initial transient increase in [K^+^]_o_ of about 0.5 mmol/L from the baseline of 3 mmol/L. This was followed by a fluctuating elevation in [K^+^]_o_ of less than 0.2 mmol/L when persistent GAM were present (Huchzermeyer et al., 2008; Kann et al., 2011; Figure 1A). In addition, repetitive electrical stimulation was superimposed on persistent GAM to get further insight into K^+^-homeostasis during fast neuronal network oscillations (Kann et al., 2011; Figure 1B). In these experiments, the amplitude of the electrically evoked [K^+^]_o_ transient did not differ in the absence (i.e., spontaneous asynchronous activity) and presence of GAM, indicating the same level of superimposed neuronal activation. By contrast, the decay time of the evoked [K^+^]_o_ transients was prolonged. This might reflect that K^+^-uptake mechanisms, such as Na^+^/K^+^-ATPase activity and glial K^+^-buffering, operate near limit during persistent GAM. Similar to GAM, sharp wave-ripples (SPW-Rs) were associated with a transient increase in [K^+^]_o_ of about 0.1 mmol/L in the CA3 network of acute hippocampal slices (Behrens et al., 2007). In this model, SPW-Rs that were induced by repetitive electrical stimulation lasted for about 70 ms, with an incidence of about 8/min (Behrens et al., 2007; Hollnagel et al., 2014). The superimposed ripples had a frequency of about 190 Hz. SPW-Rs represent a different type of fast and highly synchronous neuronal network oscillations (Draguhn et al., 1998; Maier et al., 2003; Behrens et al., 2005; Schönberger et al., 2014). They occur during consummatory behaviors and non-REM sleep and are thought to represent stored information that is transferred to, for example, the neocortex during memory consolidation (Behrens et al., 2005; Hollnagel et al., 2014; Buzsáki, 2015).

These findings provide first experimental evidence that [K^+^]_o_ indeed does not exceed the upper limit of about 3.5 mmol/L during fast neuronal network oscillations. This is in line with *in vivo* studies showing that optical stimuli transiently elevated [K^+^]_o_ by about 0.5 mmol/L in the cat visual cortex (Singer and Lux, 1975; Connors et al., 1979; Somjen, 2002). Slow neuronal network oscillations, such as sleep oscillations (<1 Hz) in the cat neocortex, were associated with periodic elevations in [K^+^]_o_ of about 1.8 mmol/L (Amzica and Steriade, 2000). These data contrast with *in vitro* and *in vivo* studies, in which repetitive electrical stimulation (up to 100 Hz, up to 60 s) was applied as a tool to activate neurons. In these studies much larger [K^+^]_o_ transients of up to 10 mmol/L from baseline were described (Heinemann and Lux, 1975, 1977; Lothman and Somjen, 1975; Gabriel et al., 1998; Kann et al., 2003b; Huchzermeyer et al., 2008). The most likely explanation is that the artificial and robust electrical stimulation evokes action potentials at unphysiological frequencies in most of the excitatory and inhibitory neurons adjacent to the stimulation electrode (Heinemann and Lux, 1977; Janigro et al., 1997)—note that during hippocampal GAM, for example, excitatory pyramidal cells and fast-spiking interneurons generate action potentials at 1–3 Hz and >30 Hz, respectively (Kann et al., 2014). Although there is depression or attenuation of spiking according to the biophysical properties of the neuronal subtype (Wong and Prince, 1981; Madison and Nicoll, 1984; Martina and Jonas, 1997; Kann et al., 2003b; Kim et al., 2012), the resulting bulk K^+^-release from neurons presumably exceeds the K^+^-uptake mechanisms in neurons and glial cells during repetitive electrical stimulation, which is reflected by the characteristic stimulus-induced [K^+^]_o_ transients. However, further experimental studies are required to determine the contribution of specific ion channels, transporters and pumps in neurons and glial cells to K^+^-homeostasis during different network activity states.

The maintenance of K^+^-homeostasis during naturally occurring fast network oscillations is likely achieved by strongly enhanced ATP generation in mitochondria—reflected by high oxygen consumption rate and near-limit utilization of oxidative capacity during GAM (see above)—to fuel ion pumps and secondary ion transport.

## Conclusion

Fast neuronal network oscillations in the gamma frequency band (30–100 Hz) occur in various regions of the cortex and have been implicated in higher brain functions such as sensory perception, attentional selection and memory formation. These GAM are associated with precise cellular adaptations to maintain energy and ion homeostasis and thus neuronal excitability and synaptic signaling. Homeostasis is apparently safeguarded by strongly enhanced oxidative phosphorylation in mitochondria to generate ATP. Conversely, metabolic stress and/or ion channel dysfunction might contribute to the exceptional vulnerability of GAM and thus higher brain functions in brain disease.

## Author Contributions

OK, J-OH, SE and JS wrote the manuscript. OK created the figures.

## Conflict of Interest Statement

The authors declare that the research was conducted in the absence of any commercial or financial relationships that could be construed as a potential conflict of interest.
